# The genome of a vestimentiferan tubeworm (*Ridgeia piscesae*) provides insights into its adaptation to a deep-sea environment

**DOI:** 10.1186/s12864-023-09166-y

**Published:** 2023-02-11

**Authors:** Muhua Wang, Lingwei Ruan, Meng Liu, Zixuan Liu, Jian He, Long Zhang, Yuanyuan Wang, Hong Shi, Mingliang Chen, Feng Yang, Runying Zeng, Jianguo He, Changjun Guo, Jianming Chen

**Affiliations:** 1 State Key Laboratory for Biocontrol, Southern Marine Science and Engineering Guangdong Laboratory (Zhuhai), School of Marine Sciences, Sun Yat-Sen University, Zhuhai, 519082 China; 2 China-ASEAN Belt and Road Joint Laboratory On Mariculture Technology, Guangdong Province Key Laboratory for Aquatic Economic Animals, School of Life Sciences, Sun Yat-Sen University, Guangzhou, 510275 China; 3 State Key Laboratory Breeding Base of Marine Genetic Resources, Key Laboratory of Marine Genetic Resources of Ministry of Natural Resources, Fujian Key Laboratory of Marine Genetic Resources, Ministry of Natural Resources, Third Institute of Oceanography, Xiamen, 361005 China; 4 Novogene Bioinformatics Institute, Beijing, 100083 China; 5 Fujian Key Laboratory On Conservation and Sustainable Utilization of Marine Biodiversity, Fuzhou Institute of Oceanography, Minjiang University, Fuzhou, 350108 China

**Keywords:** Vestimentiferan tubeworm, *Ridgeia piscesae*, Hydrothermal vent, Genome evolution, Deep-sea adaptation

## Abstract

**Background:**

Vestimentifera (Polychaeta, Siboglinidae) is a taxon of deep-sea worm-like animals living in deep-sea hydrothermal vents, cold seeps, and organic falls. The morphology and lifespan of *Ridgeia piscesae*, which is the only vestimentiferan tubeworm species found in the hydrothermal vents on the Juan de Fuca Ridge, vary greatly according to endemic environment. Recent analyses have revealed the genomic basis of adaptation in three vent- and seep-dwelling vestimentiferan tubeworms. However, the evolutionary history and mechanism of adaptation in *R. piscesae*, a unique species in the family Siboglinidae, remain to be investigated.

**Result:**

We assembled a draft genome of *R. piscesae* collected at the Cathedral vent of the Juan de Fuca Ridge. Comparative genomic analysis showed that vent-dwelling tubeworms with a higher growth rate had smaller genome sizes than seep-dwelling tubeworms that grew much slower. A strong positive correlation between repeat content and genome size but not intron size and the number of protein-coding genes was identified in these deep-sea tubeworm species. Evolutionary analysis revealed that *Ridgeia pachyptila* and *R. piscesae*, the two tubeworm species that are endemic to hydrothermal vents of the eastern Pacific, started to diverge between 28.5 and 35 million years ago. Four genes involved in cell proliferation were found to be subject to positive selection in the genome of *R. piscesae*.

**Conclusion:**

*Ridgeia pachyptila* and *R. piscesae* started to diverge after the formation of the Gorda/Juan de Fuca/Explorer ridge systems and the East Pacific Rise. The high growth rates of vent-dwelling tubeworms might be derived from their small genome sizes. Cell proliferation is important for regulating the growth rate in *R. piscesae*.

**Supplementary Information:**

The online version contains supplementary material available at 10.1186/s12864-023-09166-y.

## Introduction

The discovery of deep-sea hydrothermal vents and cold seeps, as well as their associated ecosystems, has revolutionized our view of biology and understanding of the energy sources that fuel primary productivity on Earth [1–3]. Hydrothermal vents are areas on the ocean floor where hot, anoxic, chemical-rich water is expelled into the cold, oxygen-rich deep ocean [4]. Cold seeps are areas where methane, hydrogen sulfide, and other hydrocarbons seep or emanate as gas from deep geologic sources [5]. Both hydrothermal vents and cold seeps are characterized by high hydrostatic pressure, darkness, a lack of oxygen and photosynthesis-derived nutrients, and high concentrations of toxic chemicals [6]. Organisms inhabiting around hydrothermal vents and cold seeps develop unique characters to adapt to these deep-sea reducing environments [7, 8]. Due to the complete absence of light, hydrothermal vent and cold seep ecosystems are driven by chemosynthesis instead of photosynthesis [9, 10]. The process is completed by chemosynthetic microorganisms, which cooperate with a variety of macrobenthos to form chemosynthetic symbioses and contribute to primary production supporting the ecosystem [11].

Vestimentifera (Polychaeta, Siboglinidae) is a taxon of deep-sea worm-like animals living in deep-sea hydrothermal vents, cold seeps, and organic falls [12]. The body of the adult vestimentiferan tubeworm is enclosed in a chitinous tube that is closed at the posterior end. Vestimentiferan tubeworms lack a digestive tract and rely on symbiosis with chemoautotrophic microorganisms, which inhabit a specialized internal organ, to meet their metabolic needs [13]. The first discovery of chemoautotrophic symbionts in *Riftia pachyptila*, a vestimentiferan tubeworm inhabiting hydrothermal vents on the East Pacific Rise (EPR), initiated the intensive study of these deep-sea tubeworms [1]. The body of the adult tubeworm comprises four main parts. The anteriorly located branchial plume is the primary site of gas exchange with the environment. Below the plume is the vestimentum, where the heart, gonopores and a simplified brain are located. The trophosome, which is primarily composed of symbiont-containing bacteriocytes and blood vessels, is located below the vestimentum. The segmented opisthosoma is located below the vestimentum [14–16].

Lifespan varies greatly between vent- and seep-dwelling tubeworms [17]. The lifespan of *R. pachyptila*, which thrives in relatively strong and continuous diffuse hydrothermal flow, was estimated to be less than 10 years [18]. In contrast, *Lamellibrachia luymesi*, which lives around cold seeps in the Gulf of Mexico, can live for up to 250 years [19]. In general, vent-dwelling tubeworms grow faster than seep-dwelling tubeworms. The growth rate of *R. pachyptila* can reach approximately 160 cm yr^−1^, while the growth rate of *L. luymesi* is only approximately 3 cm yr^−1^ [20–22]. A previous analysis revealed that both vent- and seep-dwelling tubeworms have high rates of cell proliferation, and the variation in growth rates is attributed to the variation in apoptosis between vent- and seep-dwelling tubeworms, where apoptosis is substantially downregulated in vent-dwelling species [23].

The Juan de Fuca Ridge in the northeast Pacific Ocean is characterized by broad heterogeneity in chemical environments, ranging from vigorous, high-temperature vents to diffuse flow [24]. The hydrothermal vents on the Juan de Fuca Ridge provide numerous biotic habitats, which support the growth of a large quantity of endemic organisms [25]. Although the biomass of the endemic hydrothermal vent fauna is high, there are only a few macrofaunal species dominating a particular vent community [26, 27]. *Ridgeia piscesae* Jones (1985) is the only vestimentiferan tubeworm species in the hydrothermal vents on the Juan de Fuca Ridge. The tubeworm occurs at high density in most vents and acts as an ecosystem-structuring species by providing habitats for several other organisms and serving as a primary producer through chemosynthetic endosymbiosis [8, 28].

*Ridgeia piscesae* adopts different strategies to thrive in diverse environments in the vents on Juan de Fuca Ridge. First, two extreme growth forms (morphotypes) of *R. piscesae*, “short-fat” and “long-skinny”, were discovered in geologically and chemically diverse vent fields [29]. The tube of the “short-fat” morphotype has a generally constant diameter of 2–3 cm, while the tube diameter of the “long-skinny” morphotype decreases from the anterior to posterior [30]. “Short-fat” *R. piscesae* prefers a relatively high-flow vent fluid of high temperature (up to 30 ℃) and high concentrations of sulfide. The “long-skinny” morphotype adapts to ambient temperature (2 ℃) and low concentrations of sulfide in areas of diffuse hydrothermal fluids [31]. This morphotype of *R. piscesae* can thrive in areas of diffuse vent fluids by acquiring sulfide using buried posterior tube sections [28]. Second, the lifespan of *R. piscesae* varies greatly according to the endemic environment [18]. The species can grow with high growth rates ranging from 6 to 95 cm yr^−1^ under favorable conditions and can grow very slowly when exposed to low levels of vent flow and sulfide [32, 33]. Strong phenotypic plasticity and a flexible lifespan allow *R. piscesae* to survive in diverse habitats and make it a unique species in the family Siboglinidae. Recent genomic analyses have revealed the genetic basis of adaptation in three vent- and seep-dwelling vestimentiferan tubeworms [34–36]. Although it plays a critical role in supporting the vent ecosystem, the evolutionary history and mechanism of adaptation in *R. piscesae*, a unique deep-sea tubeworm, remain to be investigated.

## Results

### Genome assembly and annotation

The samples of the “long-skinny” morphotype of *R. piscesae* were collected from the Cathedral deep-sea hydrothermal vent, Main Endeavor Field of the Juan de Fuca Ridge (47° 56’ N, 129° 05’ W, 2,181 m depth). Short-insert paired-end (180 bp, 300 bp and 500 bp) and long-insert mate-pair (2 kb, 5 kb, 10 kb and 15 kb) sequencing libraries were constructed and sequenced on the Illumina HiSeq 2000 platform. A total of 247.74 Gb of sequencing data was generated (Supplementary Table 1). Based on the *k*-mer distribution of 180-bp paired-end Illumina reads, the genome size was estimated to be 694.79 Mb with a heterozygosity of 1.2% (Supplementary Fig. 1). The final assembly of the *R. piscesae* genome was 574.96 Mb with a contig N50 size of 10.42 kb and a scaffold N50 size of 230.23 kb (Supplementary Table 2).

A total of 87.4% sequencing reads could be aligned unambiguously to the assembled *R. piscesae* genome sequence, covering 99.74% of the assembly (Supplementary Table 3). In addition, 99.63% of Trinity assembled sequences (unigenes) could be aligned to the assembly (Supplementary Table 4). A benchmarking universal single-copy orthologs (BUSCO) assessment of the integrity of the genome assembly against the metazoan core gene set showed that the completeness of the genome was 91.7% (90.4% complete and 1.3% fragmented) (Supplementary Table 5). These results demonstrated that the completeness of the *R. piscesae* genome is comparable to that of the previously published tubeworm genomes (Table 1) [34–36].

**Table 1:** Genome assembly statistics of four deep-sea vestimentiferan tubeworms

	*Ridgeia piscesae*	*Riftia pachyptila* [36]	*Paraescarpia echinospica* [34]	*Lamellibrachia luymesi* [35]
Assembled genome size (Mb)	574.9	560.7	1090.9	687.7
Contig N50 (kb)	10.4	2870.3	253.6	24
Scaffold N50 (kb)	230.2	-	67,235.3	372.9
BUSCO (%)	92.6	99.4	95.1	95.8
Repeat content (%)	30.2	29.9	55.1	38.2

Transposable elements (TEs) accounted for 30.17% of the *R. piscesae* genome assembly, with long interspersed elements (LINEs, 8.08%) as the most abundant class of TEs (Supplementary Table 6). The *R. piscesae* genome encodes 24,096 protein-coding genes, of which 95.54% are annotated based on known proteins in diverse public protein databases (Supplementary Table 7).

### Phylogenomic analyses

**Fig. 1:**
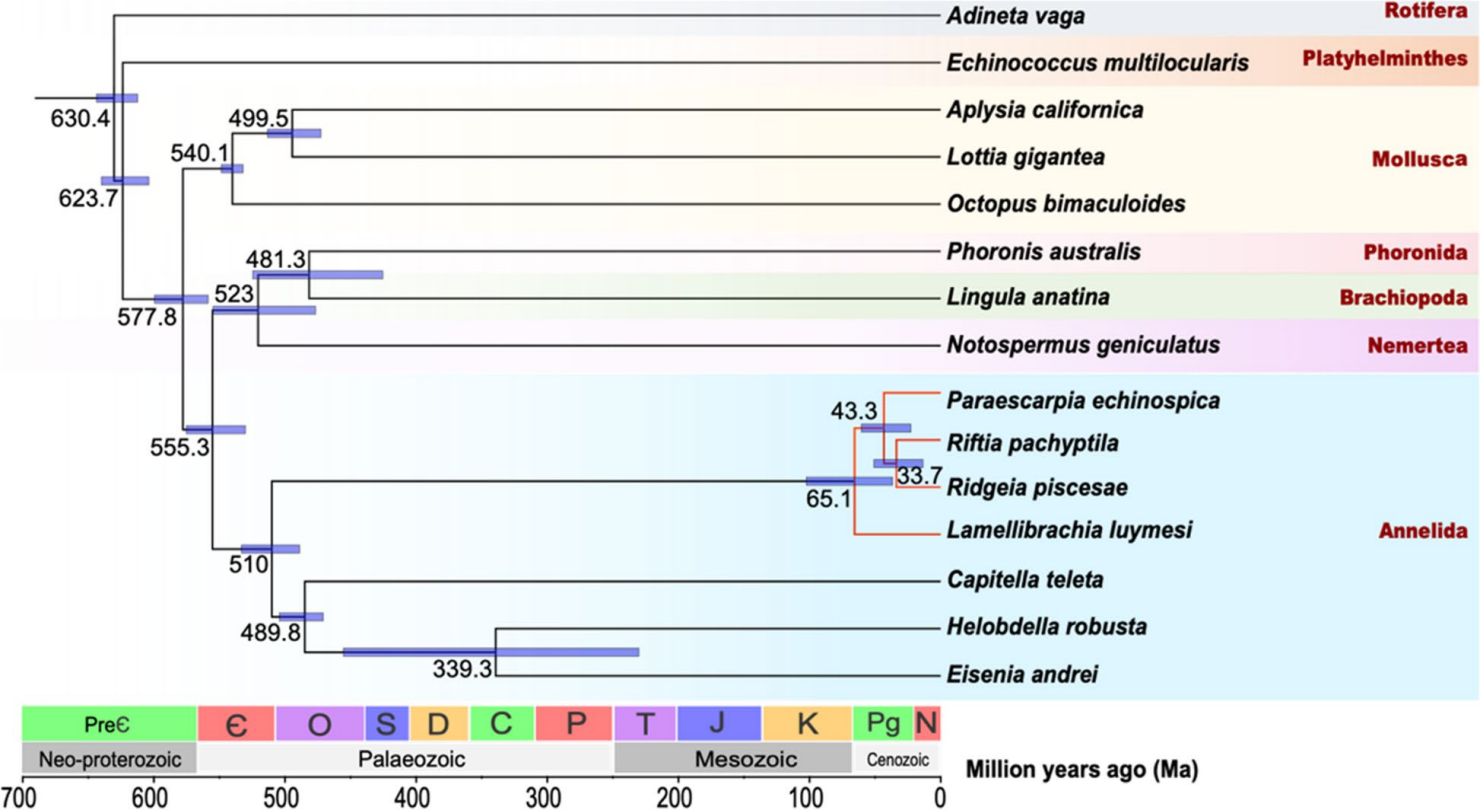
A species tree of R. piscesae and 14 metazoans. Single-copy orthologs were used to reconstruct the phylogenetic tree. The divergence time between species pairs was listed above each node, and 95% confidence interval of the estimated divergence time was denoted as blue bar. *R. piscesae* diverged from *R. pachyptila* approximately 33.7 Ma ago

To infer the evolutionary history of *R. piscesae*, a maximum-likelihood (ML) phylogenetic tree was constructed using single-copy orthologs of *R. piscesae* and 14 metazoans with *Adineta vaga* as an outgroup (Fig. 1, Supplementary Fig. 2, Supplementary Table 8). Two vent-dwelling tubeworms (*R. piscesae* and *R. pachyptila*) formed a clade. *Paraescarpia echinospica* and *L. luymesi* from cold seeps are basal to the vent clade. These results corroborate the view that vent-dwelling tubeworms might be derived from their seep-dwelling relatives [37, 38].

*Ridgeia piscesae* is endemic to the Gorda/Juan de Fuca/Explorer (GFE) ridge systems, and *R. pachyptila* is discovered on the EPR. The subduction of the Farallon-Pacific Ridge separated the GFE and EPR between 28.5 and 35 Ma ago. Molecular clock analysis revealed that the divergence time of *R. pachyptila* and *R. piscesae* was approximately 33.7 million years (Ma), suggesting that the divergence time between these two species might have been close to the separation time of the two ridge systems. The divergence time of *L. luymesi* and the other three tubeworms was estimated to be approximately 65.1 Ma, corroborating the view that modern vestimentiferan tubeworms started to diverge during the early Cenozoic Era [34, 35, 39].

### Genome evolution of vestimentiferan tubeworms

**Fig. 2:**
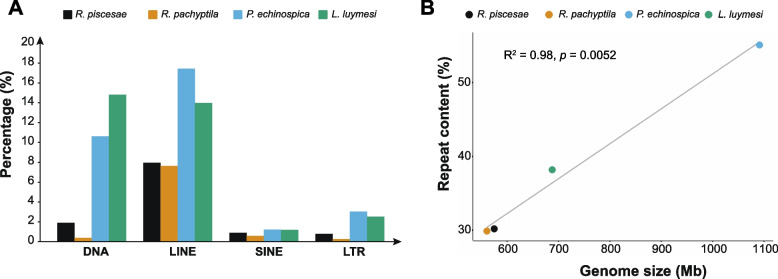
Genome size evolution in four vestimentiferan tubeworms. **A** Comparison of the occurrence and composition of repetitive elements in the genomes of 4 vestimentiferan tubeworms. **B** The relationship between repeat contents and genome sizes in 4 vestimentiferan tubeworms. A strong positive correlation (R2 = 0.98, *P* = 0.0052) was identified between genome sizes and repeat contents in these four species

**Fig. 3:**
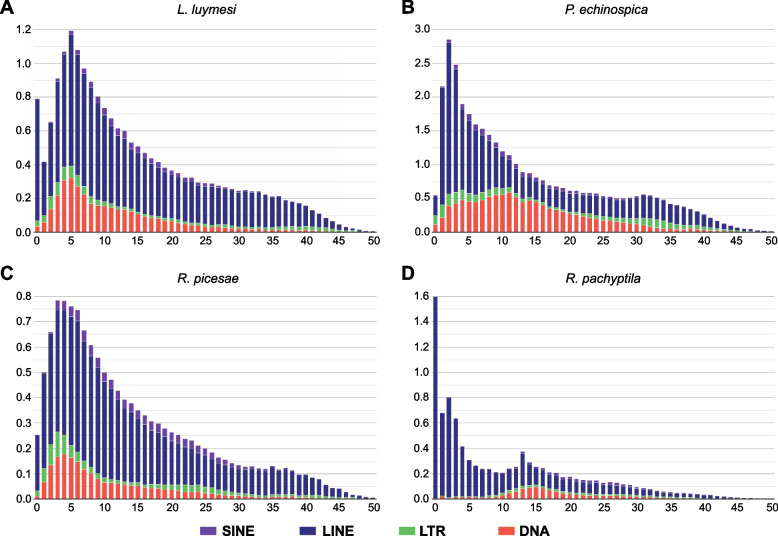
Transposable element-accumulation profile in the genomes of four vestimentiferan tubeworms. There are recent expansions of TEs in the genomes of *L. luymesi*, *P. echinospica*, and *R. piscesae*, but not in *R. pachyptila*

It has been demonstrated that several factors, including repeat content and number of genes, contribute to the variation in genome sizes among different organisms [40, 41]. In addition, previous reports proposed that the differences in genome sizes among deep-sea tubeworms might be attributable to the numbers of repetitive elements and genes [34, 36]. The assembled genome size of *R. piscesae* is similar to that of *R. pachyptila* but smaller than those of two seep-dwelling tubeworms (*L. luymesi* and *P. echinospica*) (Table 1). The genomes of cold seep-dwelling tubeworms have more TEs, especially DNA transposons, LINEs and LTR retrotransposons, than those of hydrothermal vent-dwelling tubeworms (Fig. 2a). TEs accounted for 38.2% and 55.1% of the *L. luymesi* and *P. echinospica* genomes, and they constituted 30.2% and 29.9% of the genomes of *R. piscesae* and *R. pachyptila,* respectively. A strong positive correlation (R^2^ = 0.98, *P* = 0.0052) was identified between genome size and repeat content in these four species (Fig. 2b), suggesting that TEs are a major contributor to genome size evolution in vestimentiferans. Repeat landscape plots indicated that TE activity is different between *R. pachyptila* and three other tubeworm species (Fig. 3). There are recent expansions of TEs in the genomes of *L. luymesi*, *P. echinospica*, and *R. piscesae* but not in *R. pachyptila*. The main contributors to recent TE expansions in *L. luymesi*, *P. echinospica*, and *R. piscesae* appear to have been LINEs and DNA transposons. Nonetheless, only LINEs were expanded recently in the genome of *R. pachyptila* (Fig. 3d).

The number of annotated gene models in the *R. piscesae* genome (24,096) is similar to those in the genomes of *R. pachyptila* (25,984) and *P. echinospica* (22,642) but smaller than that in the *L. luymesi* genome (38,998). Introns account for 220.1 Mb and 204.7 Mb of the genomes of two seep-dwelling tubeworms (*L. luymesi* and *P. echinospica*, respectively), as well as 264.8 Mb and 234.5 Mb of the genomes of two vent-dwelling tubeworms (*R. pachtypila* and *R. piscesae*, respectively) (Supplementary Table 9). The average length of introns in the genome of *R. piscesae* is longer than that in the genomes of the other three species. Additionally, two vent-dwelling tubeworms with smaller genome sizes had higher ratios of intron/exon length than the seep-dwelling tubeworms. Thus, gene number and intron size do not contribute to the differences in genome sizes between seep- and vent-dwelling tubeworms.

**Fig. 4:**
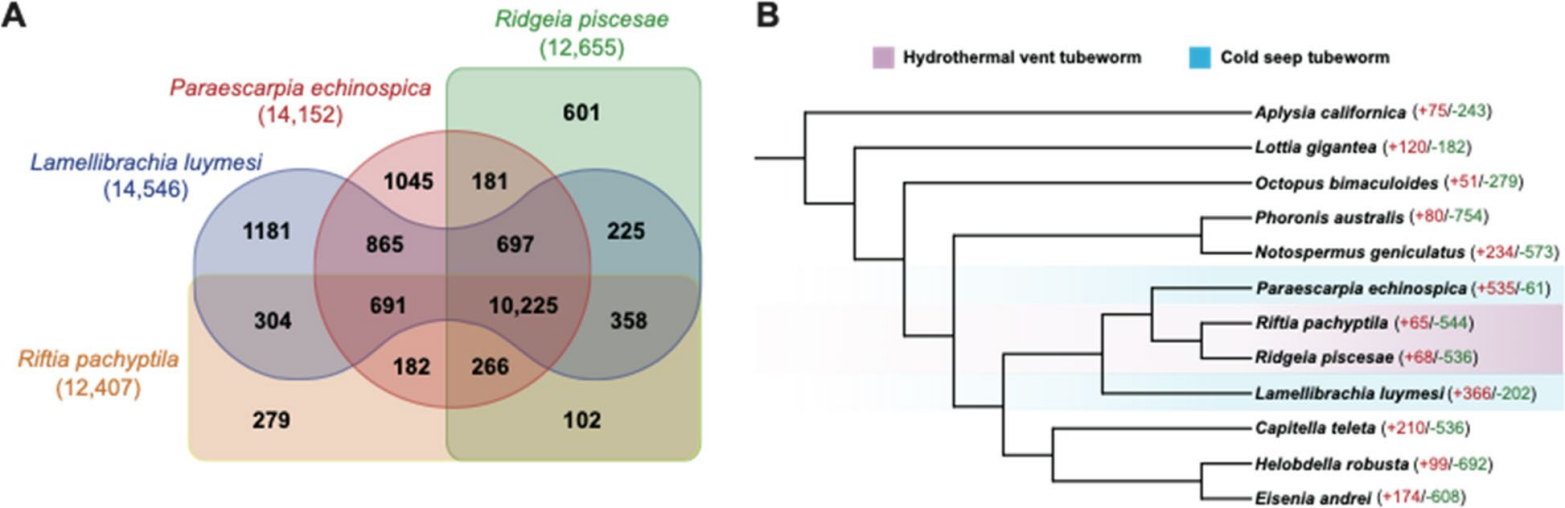
Protein family evolution in four vestimentiferan tubeworms. **A** Venn diagram of shared and unique gene families in four vestimentiferan tubeworm species. Lineage-specific gene families of *R. piscesae* and *R. pachyptila* are much less than those of *L. luymesi* and *P. echinospica*. **B** Gene family expansion/contraction analysis of 4 vestimentiferan tubeworms and 8 other lophotrochozoans. The numbers of protein families that were significantly expanded (red) and contracted (green) (*P* < 0.05) in each species are denoted beside the species names

A previous study revealed that *R. pachyptila* experienced reductive evolution with more contracted than expanded gene families in the genome [36]. Gene-family analysis of four tubeworm species identified a core set of 10,225 gene families (Fig. 4a). In total, 601 and 279 lineage-specific gene families were identified in *R. piscesae* and *R. pachyptila*, respectively, which are much smaller than the numbers in *L. luymesi* (1181) and *P. echinospica* (1045). Additionally, gene-family analysis of 12 lophotrochozoans revealed that the numbers of expanded gene families were substantially smaller than those of contracted gene families in the two vent-dwelling tubeworms, while more gene families were expanded than contracted in their seep-dwelling counterparts (Fig. 4b). These results indicate that the genomes of vent-dwelling tubeworms are characterized by gene loss.

*Hox* genes are a set of conserved regulators that specify regions of the body plan of an embryo along the anterior–posterior axis in metazoans [42]. One of the Hox genes (*Antp*) plays a role in the development of the posterior segment of several marine annelids [43]. Loss of *Antp* was apparent across all four tubeworm genomes (Supplementary Fig. 3), corroborating the view that the loss of *Antp* contributes to the reduced segmentation of the posterior region of juvenile worms in vestimentiferans [34]. The *Lox2* gene is missing from the genome of *L. luymesi* but present in the genomes of three other tubeworms, suggesting that the loss of this gene was a lineage-specific event.

### Genomic basis of deep-sea adaptation

**Fig. 5:**
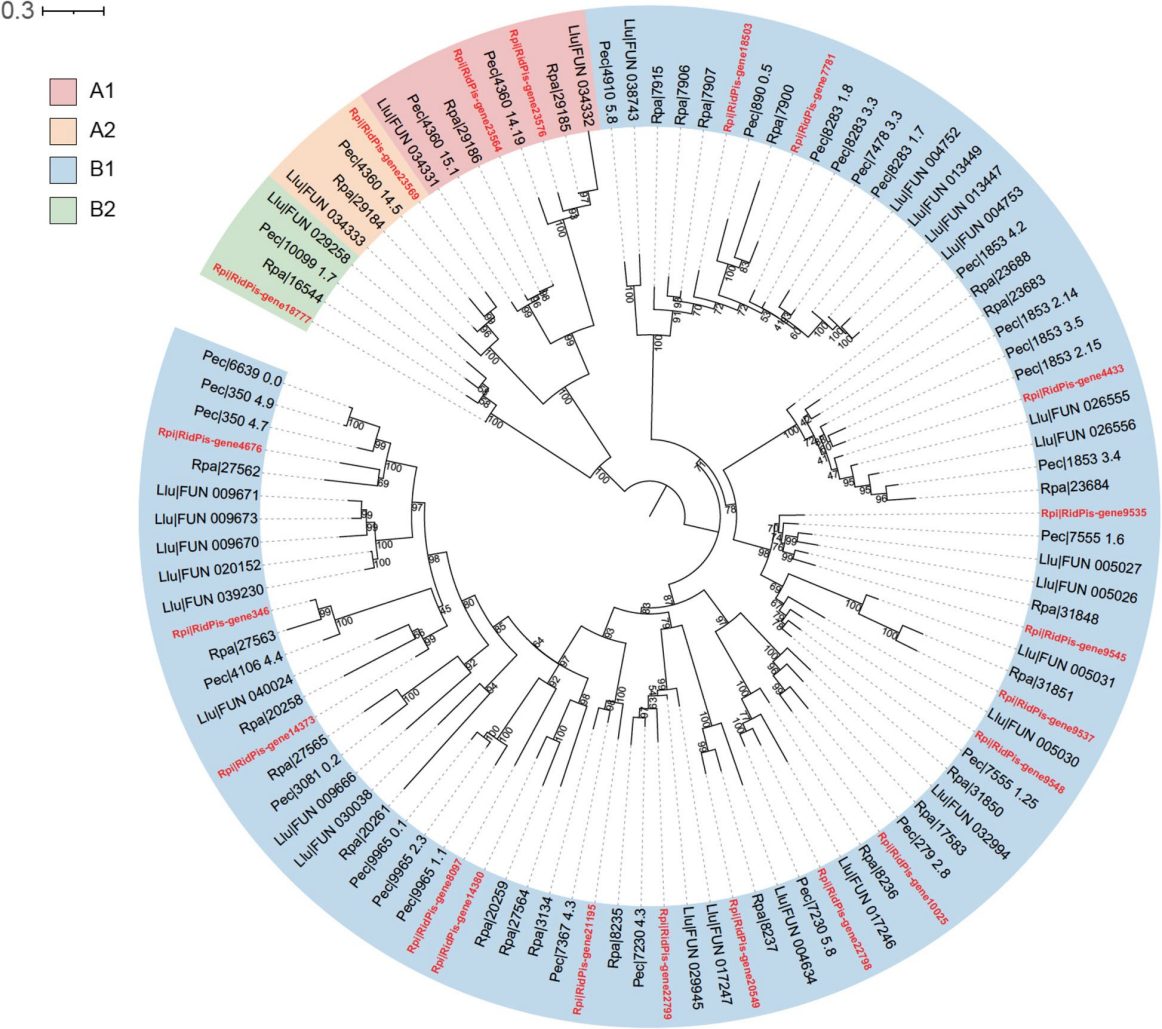
Gene tree of Hb subunits A1, A2, B1, and B2 from four vestimentiferan tubeworms. The values near the nodes are ultrafast bootstrap (UFBoot) values. Genes from *R. piscesae* are labeled in red

Hemoglobins (Hbs) in vestimentiferan tubeworms, which bind oxygen and sulfide simultaneously and provide substrate for chemosynthesis by the symbionts, facilitate the adaptation of these species to deep-sea reducing environments. Four heme-containing chains were identified (A1, A2, B1, and B2) in hemoglobins of vestimentiferans [44]. To elucidate the evolution of Hbs in vestimentiferans, we identified Hb genes in the genomes of four tubeworm species. A single copy each of A2 and B2 Hb genes, as well as two copies of A1 genes, was identified in each of the tubeworm genomes (Fig. 5). The free cysteine residues in the A2 and B2 chains contribute to the sulfide-binding ability of the vestimentiferan Hbs [45]. A2 and B2 Hb genes are highly expressed in the muscle of the vestimentum of *R. piscesae*, suggesting their role in binding H_2_S in this species (Supplementary Table 10). Previous studies revealed that the group of B1 Hbs was significantly expanded in *L. luymesi*, *P. echinospica*, and *R. pachyptila* [34–36]. With 17 identified genes, the group of B1 Hbs was also expanded in the genome of *R. piscesae*. Additionally, free cysteine was identified at the same position in 6 B1 Hb genes as in the A2 Hb genes of *R. piscesae* (Supplementary Fig. 4). The B1 genes with free cysteine are expressed in the muscle of the vestimentum of *R. piscesae*, corroborating the view that the free cysteines might also contribute to sulfide binding in the B1 hemoglobin chain of deep-sea tubeworms [35] (Supplementary Table 10). The expression levels of these B1 genes are highly variable, with only one gene highly expressed in the muscle of the vestimentum. This indicates that the vestimentum of *R. piscesae* might not be the major organ where B1 globins bind H_2_S.

**Fig. 6:**
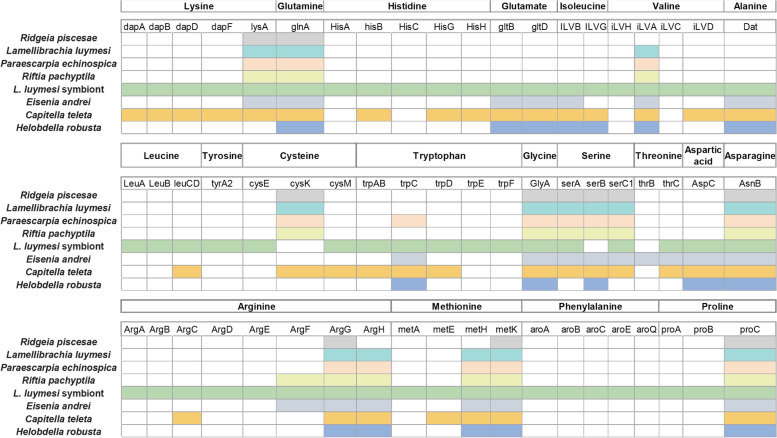
The presence and absence of key amino acid biosynthesis genes in annelids and *L. luymesi* symbiont. Most key genes associated with amino acid biosynthesis are missing in the genomes of four tubeworms (*L. luymesi*, *P. echinospica*, *R. pachyptila*, and *R. piscesae*). These genes are presented in the genome of L. luymesi symbionts. Two other annelids (*E. andrei* and *H. robusta*) that acquire amino acids from food also lack some key genes associated with amino acid biosynthesis

Recent reports revealed that most enzymes related to amino acid biosynthesis were lost in *L. luymesi* and *R. pachyptila* [35, 36]. To gain better insight into the nutrient dependence of endosymbionts in vestimentiferans, we identified key enzymes involved in amino acid biosynthesis in the genomes of 4 tubeworms and 3 other annelid species (Fig. 6). All four tubeworms (*L. luymesi* and *R. pachyptila*, *R. piscesae* and *P. echinospica*) lack most key enzymes related to amino acid biosynthesis, corroborating the view that vestimentiferan tubeworms mainly rely on endosymbionts for synthesizing amino acids [35]. In addition to tubeworms, two other annelids (*Eisenia andrei* and *Helobdella robusta*) also lack most enzymes for amino acid biosynthesis, supporting the hypothesis that these two species acquire amino acids from food [46, 47].

The expansion of gene families is considered a major driver of adaptation and speciation [48]. Thus, we performed gene family expansion and contraction analysis with 4 vestimentiferan tubeworms and 8 other lophotrochozoans (Fig. 4b). In total, 10 gene families were significantly expanded in the genomes of all four tubeworms compared to the other 8 lophotrochozoans (*P* < 0.05) (Supplementary Table 11). Gene ontology analysis revealed that the expanded gene families were involved in chitin binding and innate immunity. Furthermore, 18 gene families were significantly expanded in the genomes of two cold-seep tubeworms. The expanded gene families were involved in DNA repair, innate immunity, and protein stability (Supplementary Table 12).

In addition to expanded and contracted gene families, we also identified positively selected genes (PSGs) in the genome of *R. piscesae*. Compared with 11 other lophotrochozoan species, *R. piscesae* was found to harbor 9 PSGs. In total, 7 out of 9 PSGs were expressed in the vestimentum of *R. piscesae* (Supplementary Table 13). Interestingly, four genes (alkB homolog 2, alpha-ketoglutarate-dependent dioxygenase, *ALKBH2*; Derlin-1, *DERL1*; Ras-related and estrogen-regulated growth inhibitor, *RERG*; and AN1-type zinc finger protein 2B, *ZFAND2B*) involved in cell proliferation were subjected to positive selection in *R. piscesae*. Two of these genes (*ALKBH2* and *DERL1*) promote cell proliferation. ALKBH2 is responsible for protecting the genome from 1-meA damage by repairing the damage in double-stranded DNA [49]. *ALKBH2* promotes cell proliferation and is overexpressed in several types of tumor cells [50]. DERL1 participates in the endoplasmic reticulum (ER)-associated degradation response and unfolded protein response (UPR) [51]. *DERL1* is responsible for cell proliferation and promotes the progression of several types of cancers [52]. Interestingly, two other genes (*RERG* and *ZFAND2B*) inhibit cell proliferation. Overexpression of *RERG*, a member of the RAS superfamily of GTPases, inhibits cell proliferation and tumor formation [53, 54]. ZFAND2B reduces the abundance of IGF1R, a kinase that activates cell proliferation, in a proteasome-dependent manner [55]. These results suggest that, in addition to apoptosis, the regulation of cell proliferation also contributes to the variation in growth rates in *R. piscesae*.

## Discussion

In many hydrothermal vent and cold seep ecosystems, vestimentiferan tubeworms are among the dominant megafauna in habitats where hydrogen sulfide is present [56, 57]. *Ridgeia piscesae* is an ecosystem-structuring species and primary producer in the hydrothermal vents on the Juan de Fuca Ridge, where the biomass of the endemic fauna is high [8, 28]. In addition, *R. piscesae* has strong phenotypic plasticity, with two extreme morphotypes (“short-fat” and “long-skinny”) diverging in several morphological characters found in this species. The “short-fat” morphotype prefers relatively high-flow vent fluid with high concentrations of sulfide, while the “long-skinny” morphotype survives in diffuse hydrothermal fluids with low concentrations of sulfide [31]. Strong phenotypic plasticity makes *R. piscesae* a unique species in the family Siboglinidae. In this study, we assembled and annotated a draft genome sequence of *R. piscesae* collected at the Cathedral vent of the Juan de Fuca Ridge.

*Riftia pachyptila* and *R. piscesae* are two tubeworm species endemic to hydrothermal vents in the eastern Pacific. *Ridgeia piscesae* and *R. pachyptila* are endemic to the GFE ridge systems and the EPR, respectively, which are separated due to the subduction of the Farallon-Pacific Ridge between 28.5 and 35 Ma. Phylogenomic analysis showed that the divergence time of *R. pachyptila* and *R. piscesae* was approximately 33.7 Ma, suggesting that the phenotypic divergence between these two species might be derived from adaptation to the local environments of the two ridge systems.

Genome sizes vary greatly among vent- and seep-dwelling tubeworms. It was proposed that natural selection and adaptive processes shape genome size evolution [58]. Previous analyses revealed that genome sizes are correlated with several phenotypic traits, including cell size and rates of metabolism and growth [59–62]. Thus, we studied the evolution of genome sizes in four vestimentiferan tubeworms. A strong positive correlation (R^2^ = 0.98, *P* = 0.0052) between repeat content and genome size was identified in these tubeworm species. Among the four vestimentiferan tubeworm genomes, the *L. luymesi* genome has the most annotated gene models, while another seep-dwelling tubeworm (*P. echinospica*) genome has the fewest annotated gene models. In addition, the average length of introns in the genome of *R. piscesae* is also longer than that in the introns of the other three species’ genomes. This suggests that repeat content contributes to the variation in genome sizes in tubeworm species. However, the variation in genome sizes in tubeworms is not attributable to gene number and intron length, as proposed in a previous study [36].

Vent-dwelling tubeworms grow much faster than seep-dwelling tubeworms. The growth rates of *R. pachyptila* and *R. piscesae* can reach approximately 160 cm yr^−1^ and 95 cm yr^−1^, respectively, while the growth rate of *L. luymesi* is only approximately 3 cm yr^−1^ [20–22, 32, 33]. We studied the factors that might contribute to the variation in growth rate in four vestimentiferan tubeworms. The lineage-specific gene families of *R. piscesae* (601) and *R. pachyptila* (279) are much smaller than those of *L. luymesi* (1181) and *P. echinospica* (1045). In addition, gene-family expansion and contraction analysis revealed that the numbers of expanded gene families were substantially smaller than those of contracted gene families in the two vent-dwelling tubeworms. A negative correlation between genome size and growth rate was identified in several species, as organisms with smaller genomes might undergo more rapid replication of their genome [62, 63]. A recent report showed that *R. pachyptila* underwent reductive evolution [36]. Our results indicate that both *R. pachyptila* and *R. piscesae* experienced reductive evolution. The small genome size of vent-dwelling tubeworms might contribute to their fast growth rates.

Previous immunohistochemical and ultrastructural cell cycle analyses revealed that both *L. luymesi* and *R. pachyptila* had extremely high cell proliferation activities. The divergence of growth rates between seep- and vent-dwelling tubeworms is attributable only to apoptosis. *Lamellibrachia luymesi* has balanced activities of proliferation and apoptosis in the epidermis, while apoptosis is substantially downregulated in this tissue in *R. pachyptila* [23]. Unlike those of other vestimentiferan tubeworms, the growth rates of *R. piscesae* vary greatly among individuals living in environments with different levels of vent flow and sulfide [32, 33]. Four genes involved in the regulation of cell proliferation were identified to be positively selected in *R. piscesae*. Interestingly, two of these genes promote cell proliferation, whereas the two other genes inhibit cell proliferation. This result indicates that both cell proliferation and apoptosis are involved in the regulation of growth in *R. piscesae*.

There is still room for improvement of *R. piscesae* genome (contig N50: 10.42 kb, scaffold N50: 230.23 kb), which may affect some genomic analyses, including underestimating the gene and repeat contents. The BUSCO estimate of the completeness of our assembly (91.7%) is comparable to that for previously published lophotrochozoan genomes. Additionally, 99.63% of Trinity assembled sequences could be aligned to our assembly. Thus, our assembly should be useful for exploring the genetic basis of deep-sea adaptation in this species. With two extreme morphotypes (“short-fat” and “long-skinny”) adapted to different environments, *R. piscesae* is a unique species in the family Siboglinidae. It will be interesting to reveal the molecular basis of these two morphotypes adapt to their endemic environments through comparative transcriptomic analysis. Unfortunately, it is limited by the sample collection from deep-sea environments. We hope to improve the quality of genome assembly and perform comparative transcriptomic analysis between the two morphotypes in future studies.

## Conclusions

Here, we assembled and annotated a draft genome of *R. piscesae* collected at the Cathedral vent of the Juan de Fuca Ridge. Evolutionary analysis suggested that the divergence between two vent-dwelling species (*R. piscesae* and *R. pachyptila*) might have been close in time to the separation of the GFE ridge systems and the EPR. Comparative genomic analysis showed that vent-dwelling tubeworms with a higher growth rate had smaller genome sizes than seep-dwelling tubeworms that grow much slower, suggesting that the high growth rates of vent-dwelling tubeworms are derived from their small genome sizes. The variation in the genome sizes of these deep-sea tubeworms is attributed to the repeat content but not the intron sizes and numbers of protein-coding genes. Finally, four genes involved in cell proliferation were found to be subject to positive selection in the genome of *R. piscesae*, indicating that cell proliferation is important for regulating the growth rate in this species.

## Methods

### Sampling and sequencing

The samples of “long-skinny” morphotype of *R. piscesae* were obtained during *Alvin* dive 4243 from the deep-sea hydrothermal vent at Cathedral vent, Main Endeavor Field of the Juan de Fuca Ridge (47° 56’ N, 129° 05’ W, 2,181 m depth) on August 9, 2006. Genomic DNA (gDNA) was extracted from vestimentum muscle of the specimen using a standard phenol/chloroform extraction protocol and broken into random fragments for whole-genome shotgun (WGS) sequencing. Agarose gel electrophoresis was used to check the quality of the gDNA, and Qubit system was used to quantify the gDNA. Short-insert paired-end libraries (180 bp, 300 bp and 500 bp) were prepared using the NEBNext Ultra DNA Library Prep Kit for Illumina (NEB, USA) according to the standard protocol, respectively. Large-insert mate-pair libraries (2 kb, 5 kb, 10 kb and 15 kb) were prepared following the Cre-lox recombination-based protocol [64]. All DNA libraries were subjected to paired-end sequencing on the Illumina Hiseq 2000 platform (Illumina). Muscle samples from vestimentum were also collected for constructing RNA sequencing (RNA-seq) library. Total RNA was extracted with TRIzol reagent (Molecular Research Center, USA). Paired-end library for RNA-seq was constructed using the Paired-End Sample Preparation Kit (Illumina Inc., San Diego, CA, USA) and sequenced on the Illumina Hiseq 2000 platform (Illumina).

### Genome assembly

NGS QC toolkit (v2.1) [65] was used to evaluate the quality of raw sequencing reads and filter high-quality reads. High-quality reads were obtained by filtering out the following types of reads: (1) reads with >  = 10% unidentified nucleotides (N); (2) reads with adaptor contamination; (3) reads with >  = 20% bases having Phred quality score <  = 5; (4) duplicated reads generated by PCR amplification during the library construction process.

The size and heterozygosity of *R. piscesae* genome were estimated using the high-quality short-insert paired-end reads (180 bp) by *k*-mer frequency-distribution method. The number of *k*-mers and the peak depth of *k*-mer sizes at 19 was obtained using GenomeScope2 (v1.0.0) [66]. Due to the high heterozygosity of *R. piscesae* genome, a modified version of SOAPdenovo [67] was implemented for genome assembly. In brief, all short-insert paired-end reads were applied for contig assembly. Depth of coverage was obtained for each contig using SOAPdenovo with the parameters ‘-e 1 -M 0 -R’, and the contigs with depth less than 60 were identified as heterozygous contigs. All WGS reads were aligned to the heterozygous contigs using SOAPdenovo. And links were generated between heterozygous contigs when supported by a minimum of three read pairs. Heterozygous contigs were clustered into bubble clusters based on the orientation and distance between heterozygous contigs. If two contigs represented two potential haplotypes in a bubble structure, the longer one was retained to ensure the integrity of contig assembly.

To scaffolding the contigs, all short-insert paired-end and long-insert mate-pair reads were realigned onto the contig sequences using SOAPdenovo. Duplicated contigs that had high depth of coverage and conflicting connections to the unique contigs were masked during scaffolding. A hierarchical assembly strategy was used to construct contigs into primary scaffolds by adding the ascending insert size reads gradually. Finally, all short-insert reads were realigned onto the scaffold sequences to fill the gaps with the GapCloser program implemented in SOAPdenovo [68].

We also attempted to assemble the genome using two other assemblers (ABySS2, Platanus-Allee) [69, 70], but the genome quality and completeness were inferior to the SOAPdenovo assembly (Supplementary Table 14). Thus, we ignore the assemblies in the downstream analysis.

### Genome quality assessment

To assess the completeness of the *R. piscesae* genome, high-quality short-insert paired-end reads were mapped to the genome assembly using Burrows-Wheeler Aligner (BWA) (v0.7.17) [71] with parameters of ‘-o 1 -e 5 -t 8 -n 15’. In addition, all RNA-seq reads were de novo assembled using Trinity (v2.9) [72]. The Trinity unigene assembled sequences with length >  = 500 bp were mapped to the *R. piscesae* genome using BLAT (v35.1) [73] with default parameters and an identify cutoff of 90%. The completeness of the assembly was also evaluated using benchmarking universal single-copy orthologs (BUSCO) (v3.1.0) [74] with 978 metazoa single-copy orthologous genes (obd10).

### Genome annotation

Tandem repeats in the genome were predicted using the program Tandem Repeats Finder (TRF) (v4.09) [75] with default parameters. Transposable elements (TEs) were identified using the homology-based and de novo prediction approaches. For homology-based prediction, RepeatMasker (v4.1.0) (http://www.repeatmasker.org/) were conducted to identify repeat sequences against the Repbase library. For de novo prediction, RepeatModeler (v2.0.1) (http://repeatmasker.org/RepeatModeler.html), LTR-Finder (v1.0.7) [76], RepeatScout (v1.0.5) [77] and Piler (v1.0) [78] were used to construct de novo repeat libraries. RepeatMasker (v4.1.0) was run against these libraries to search repeat elements.

Protein-coding genes in *R. piscesae* genome were predicted with three approaches: homology-based prediction, ab initio prediction and RNA-seq-based prediction. Protein-coding sequences of *Lottia gigantea*, *Helobdella robusta*, *Capitella teleta*, *Schistosoma mansoni*, *Caenorhabditis elegans*, *Anopheles gambiae*, *Drosophila melanogaster* and *Homo sapiens* were aligned to the *R. piscesae* genome using tblastn with a cut off E-value of 1e-5. GeneWise (v2.4) [79] was employed to predict gene models. For ab initio prediction, Augustus (v3.3.2) [80], Genscan [81], Geneid (v1.3) [82], GlimmerHMM (v3.0.4) [83] and SNAP [84] were used to predict genes on the repeat-masked genome. For RNA-seq-based prediction, unigenes generated using Trinity (v2.9) were aligned against the genome assembly with BLAT (v35.1) (identify >  = 0.95 and align rate >  = 0.95) [73]. In addition, the RNA-seq reads from were aligned to the *R. piscesae* genome using Tophat (v2.1.1) [85]. And gene structures were predicted using Cufflinks (v2.2.1) [86]. EvidenceModeler (EVM) (v1.1.1) [87] was used to integrate all gene models derived from these three approaches into a non-redundant gene set.

Functional annotation was performed using BLASTP searches against SwissProt and TrEMBL databases [88] with a E-value cut-off of 1e-5. In addition, InterProScan (v5.4.0) [89] was used to screen proteins against five databases (Pfam, PRINTS, PROSITE, ProDom and SMART) to determine protein domains and motifs. Gene Ontology (GO) annotation of each gene was retrieved from the corresponding InterPro entry. In addition, KEGG annotation was performed using GhostKOALA [90].

### Phylogenomic analysis

Protein sequences of 14 metazoan species (*Adineta vaga*, *Echinococcus multilocularis*, *Aplysia californica*, *Lottia gigantea*, *Octopus bimaculoide*, *Phoronis australis*, *Lingula anatina*, *Notospermus geniculatus*, *Capitella teleta*, *Helobdella robusta*, *Eisenia Andrei*, *Riftia pachyptila*, *Paraescarpia echinospica*, *Lamellibrachia lumysi*) were downloaded for gene family cluster analysis (Supplementary Table 8). The longest transcripts of each gene (more than 30 amino acids) were retained. All-to-all BLASTP was used to identify the similarities between retained protein sequences of these 14 metazoan species and *R. piscesae* (E-value threshold: 1e-7). OrthoFinder (v2.2.7) [91] was used to identify and cluster gene families among 15 species with default parameters. Gene clusters with > 100 gene copies in one or more species were removed. Protein sequences of all single-copy gene families were retrieved and aligned using MAFFT (v7.271) [92]. The alignments were trimmed using TrimAl (v1.2) [93]. The phylogenetic tree was reconstructed with the trimmed alignments using FastTree2 (v2.1.11) [94] with *Adineta vaga* as outgroup.

To estimate the divergent time, the trimmed alignments of single-copy orthologs among the 15 metazoan species were concatenated using PhyloSuite (v1.2.2) [95]. MCMCtree module of the PAML package (v4.9) [96] was used to estimate the divergent time with the concatenated alignment. The species tree of the 15 metazoan species was used as a guide tree, and the analysis was calibrated with the divergent time obtained from TimeTree database (minimum = 470.2 Ma and soft maximum = 531.5 Ma between *P. australis* and *L. anatina*) [97] and previous analyses (minimum = 470.2 Ma and soft maximum = 531.5 Ma between *A. californica* and *L. gigantea*; minimum = 532 Ma and soft maximum = 549 Ma for the first appearance of Mollusca; minimum = 476.3 Ma and soft maximum = 550.9 Ma for the appearance of capitellid-leech clade; minimum = 550.25 Ma and soft maximum = 636.1 Ma for the first appearance of Lophotrochozoa and Ecdysoa) [98–100].

### Gene family expansion and contraction analysis

R8s (v1.7) was applied to obtain the ultrametric tree of 12 lophotrochozoan species (*C. teleta*, *H. robusta*, *E. andrei*, *L. gigantea*, *A. californica*, *N. geniculatus*, *A. californica*, *P. australis*, *R. pachyptila*, *P. echinospica*, *L. lumysi*, *R. piscesae*), which is calibrated with the divergent time between *C. teleta* and *L. gigantea* (688 Ma) obtained from TimeTree database. CAFÉ (v5) [101] was applied to determine the significance of gene-family expansion and contraction among 12 lophotrochozoan species based on the ultrametric tree and the gene clusters determined by OrthoFinder (v2.2.7). Gene families that were significantly expanded in each of four tubeworm species (*R. pachyptila*, *P. echinospica*, *L. lumysi*, *R. piscesae*) (*P* < 0.05) were annotated using PANTHER (v16.0) with the PANTHER HMM scoring tool (pantherScore2.pl) [102].

### Homeobox gene analysis

Homeodomain sequences, which were retrieved from HomeoDB database [103], were aligned to *R. piscesae* genome assembly using tbalstn. Sequences of the candidate homeobox genes were extracted based on the alignment results. The extracted sequences were aligned against NCBI NR and HomeoDB database to classify the homeobox genes.

### Hemoglobin gene family analysis

Protein sequences of hemoglobin A1, A2, B1, B2 chains of four tubeworm species were obtained with reference references using DIAMOND BLASTP [104] with a E-value cut-off of 1e-5. The sequences were annotated in NCBI NR database using BLASTP. And protein domains in these sequences were annotated by Pfamscan against Pfam-A.hmm database [105]. Sequences that have almost full length protein domains were aligned using MAFFT (v7.271) [106]. The alignments were trimmed using TrimAI (v1.2) [93]. The phylogenetic tree was reconstructed with the trimmed alignments using a maximum-likelihood method implemented in IQ-TREE2 (v2.1.2) [107]. The best-fit substitution model was selected by using ModelFinder algorithm [108]. Branch supports were assessed using the ultrafast bootstrap (UFBoot) approach with 1,000 replicates [109].

### Identification of positively selected genes (PSGs)

We identified PSGs in the *R. piscesae* genome within single-copy orthologs among 12 lophotrochozoan species that were identified in gene-family expansion and contraction analysis. Protein sequences of all single-copy gene families were retrieved and aligned using MAFFT (v7.271) [92]. Phylogenetic tree of each family was reconstructed using IQ-TREE2 (v2.1.2) [107]. PSGs were identified based on the phylogenetic trees using HyPhy (v2.5.30) with the adaptive Branch-Site Random Effects Likelihood (aBSREL) model [110].

### Statistics and reproducibility

Alpha levels of 0.05 were regarded as statistically significant throughout the study, unless otherwise specified.

## Supplementary Information

**Additional file 1:** **Supplementary Figure 1.** Distributionof 19-mer frequency in *Ridgeia piscesae*genome. The short-insert paired-end reads (180 bp) were used to generate the19-mer frequency curve. Theheterozygous rate and the genome size were determined based on the *k*-merdistribution. **Supplementary Figure 2.** The phylogenetic tree of *R.piscesae* and 14 other lophotrochozoans.The tree wasreconstructed with single-copy orthologs using a maximum likelihood approach.The ultrafast bootstrap (UFBoots) value is listed above each of the nodes.**Supplementary Figure 3.** Genomic organization of *Hox* gene clusters in 4vestimentiferan tubeworms and 11 other metazoans. *Hox* genes are indicated as rectangles.The orientations of genes are indicated by arrows below the genes. The genecomposition and orientation of *Hox* clusters are consistent between two vent-dwellingtubeworms (*R. pachyptila* and *R. piscesae*), but slightly differentbetween vent- and seep-dwelling tubeworms. **Supplementary Figure 4.**Alignment of hemoglobins in four tubeworms. Each tubeworm has two copies of A1 chain, one copy of A2 chain, and onecopy of B2 chain in hemoglobins of tubeworms. A group of B1 chain in hemoglobinwere found in each of four species. Free cysteine was found in A2, B2, and B1chains in hemoglobin. **Supplementary Table 1.** Statistics of the genome sequencing data of *Ridgeia piscesae*. **Supplementary Table 2.**  Statistic of the *R. piscesae* genome assembly. **Supplementary Table 3.**  Assessment of genome coverage ratebased on short-insert paired-end reads remapping analysis. **SupplementaryTable 4.**  Assessment of gene coveragerate using Trinity assembled sequences (Unigenes). **SupplementaryTable 5.**  BUSCO evaluationof *R. piscesae* genome assembly. **Supplementary Table 6.**  Summary of annotated repeats in *R.piscesae* genome. **Supplementary Table 7.**  Statistics of functional annotated gene models in the genome of *R.piscesae*. **Supplementary Table 8.**  Information of genomes used to perform phylogenomic analysis. **Supplementary Table 9.**  Exon and intronlengths of genes in four Vestimentiferan tubeworms. **Supplementary Table 10.**  Expression levels of hemoglobin genes withfree cysteine in *R. piscesae*. **Supplementary Table 11.**  Gene families were significantly expandedin the genomes of all four tubeworms. **SupplementaryTable 12.**  Gene families were significantly expanded in the genomes of twoseep-dwelling tubeworms. **Supplementary Table 13.**  Positively selected genes (PSGs) in *R.piscesae*. **Supplementary Table 14.**  Summary of genome assemblies using two other assemblers

## Data Availability

Raw reads and genome assembly are accessible in NCBI under BioProject number PRJNA826206. Raw reads and genome assembly are also available at the CNGB Sequence Archive (CNSA) of China National GeneBank DataBase (CNGBdb) with accession number CNP0002911.

## Abbreviations

EPR: East Pacific Rise
GFE: Gorda/Juan de Fuca/Explorer
BUSCO: Benchmarking universal single-copy orthologs
TEs: Transposable elements
LINE: Long interspersed element
ML: Maximum likelihood
Ma: Million years
Hb: Hemoglobin
PSG: Positively selected gene
ALKBH2: AlkB homolog 2, alpha-ketoglutarate-dependent dioxygenase
DERL1: Derlin-1
RERG: Ras-related and estrogen-regulated growth inhibitor
ZFAND2B: AN1-type zinc finger protein 2B
UPR: Unfolded protein response
