# Supplementary material for: The genome of a vestimentiferan tubeworm (Ridgeia piscesae) provides insights into its adaptation to a deep-sea environment
Source: BMC Genomics. 2023 Feb 11;24:72. doi: 10.1186/s12864-023-09166-y (PMC9921365; doi:10.1186/s12864-023-09166-y)
Supplement: Supplementary file 1 — Additional file 1: Supplementary Figure 1. Distributionof 19-mer frequency in Ridgeia piscesaegenome. The short-insert paired-end reads (180 bp) were used to generate the19-mer frequency curve. Theheterozygous rate and the genome size were determined based on the k-merdistribution. Supplementary Figure 2. The phylogenetic tree of R.piscesae and 14 other lophotrochozoans.The tree wasreconstructed with single-copy orthologs using a maximum likelihood approach.The ultrafast bootstrap (UFBoots) value is listed above each of the nodes.Supplementary Figure 3. Genomic organization of Hox gene clusters in 4vestimentiferan tubeworms and 11 other metazoans. Hox genes are indicated as rectangles.The orientations of genes are indicated by arrows below the genes. The genecomposition and orientation of Hox clusters are consistent between two vent-dwellingtubeworms (R. pachyptila and R. piscesae), but slightly differentbetween vent- and seep-dwelling tubeworms. Supplementary Figure 4.Alignment of hemoglobins in four tubeworms. Each tubeworm has two copies of A1 chain, one copy of A2 chain, and onecopy of B2 chain in hemoglobins of tubeworms. A group of B1 chain in hemoglobinwere found in each of four species. Free cysteine was found in A2, B2, and B1chains in hemoglobin. Supplementary Table 1. Statistics of the genome sequencing data of Ridgeia piscesae. Supplementary Table 2. Statistic of the R. piscesae genome assembly. Supplementary Table 3. Assessment of genome coverage ratebased on short-insert paired-end reads remapping analysis. SupplementaryTable 4. Assessment of gene coveragerate using Trinity assembled sequences (Unigenes). SupplementaryTable 5. BUSCO evaluationof R. piscesae genome assembly. Supplementary Table 6. Summary of annotated repeats in R.piscesae genome. Supplementary Table 7. Statistics of functional annotated gene models in the genome of R.piscesae. Supplementary Table 8. Information of genomes used to perform phylogenomic analysis. Supplementary Table 9. Ex [file 12864_2023_9166_MOESM1_ESM.docx]

**Supplementary Information for**

***The genome of a vestimentiferan tubeworm* (*Ridgeia piscesae*) *provides insights into its adaptation to a deep-sea environment***


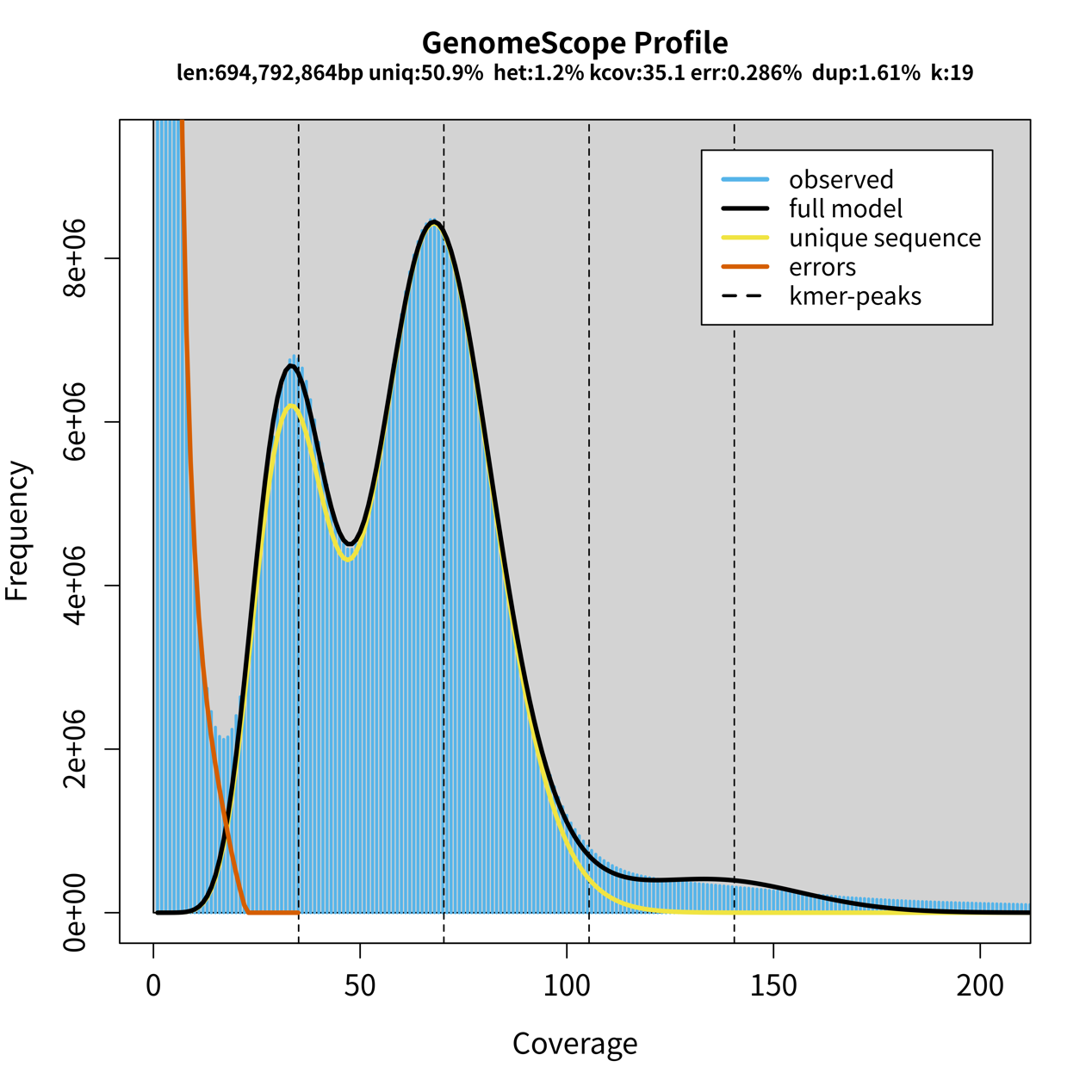


**Supplementary Figure 1. Distribution of 19-mer frequency in *Ridgeia piscesae* genome.** The short-insert paired-end reads (180 bp) were used to generate the 19-mer frequency curve. The heterozygous rate and the genome size were determined based on the *k*-mer distribution.

**Supplementary Figure 2. The phylogenetic tree of *R. piscesae* and 14 other lophotrochozoans.** The tree was reconstructed with single-copy orthologs using a maximum likelihood approach. The ultrafast bootstrap (UFBoots) value is listed above each of the nodes.

**Supplementary Figure 3. Genomic organization of *Hox* gene clusters in 4 vestimentiferan tubeworms and 11 other metazoans.** *Hox* genes are indicated as rectangles. The orientations of genes are indicated by arrows below the genes. The gene composition and orientation of *Hox* clusters are consistent between two vent-dwelling tubeworms (*R. pachyptila* and *R. piscesae*), but slightly different between vent- and seep-dwelling tubeworms.

**~~
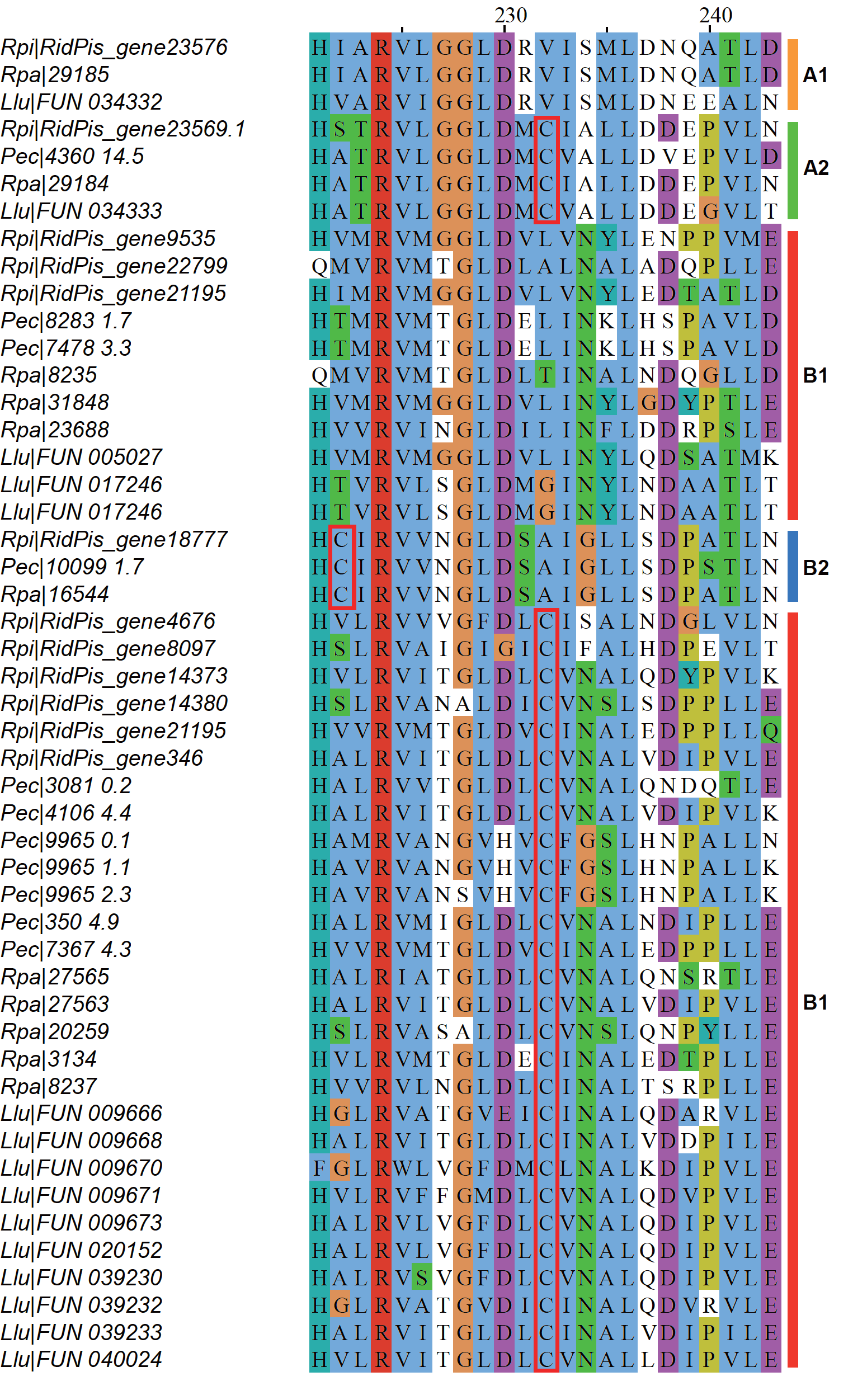
~~**

**Supplementary Figure 4. Alignment of hemoglobins in four tubeworms.** Each tubeworm has two copies of A1 chain, one copy of A2 chain, and one copy of B2 chain in hemoglobins of tubeworms. A group of B1 chain in hemoglobin were found in each of four species. Free cysteine was found in A2, B2, and B1 chains in hemoglobin.

**Supplementary Table 1.** Statistics of the genome sequencing data of *Ridgeia piscesae*

| **Pair-end  libraries** | **Insert size** | **Raw data (Gb)** | **Read length (bp)** | **Sequence coverage (X)** † |
| --- | --- | --- | --- | --- |
| Illumina data | 180 bp | 87.13 | 100 | 125.40 |
|  | 300 bp | 40.62 |  | 58.46 |
|  | 500 bp | 38.74 |  | 55.76 |
|  | 2 kb | 18.73 |  | 26.96 |
|  | 5 kb | 20.76 |  | 29.88 |
|  | 10 kb | 31.85 |  | 45.84 |
|  | 15 kb | 9.91 |  | 14.26 |
| Total | - | 247.74 | - | 356.56 |

†Sequencing coverage was calculated with the clean data and the estimated genome size of 694.79 Mb by *k*-mer analysis

**Supplementary Table 2.** Statistic of the *R. piscesae* genome assembly

| **Sample ID** | **Contig** | | **Scaffold** † | |
| --- | --- | --- | --- | --- |
|  | **number** | **Length** | **number** | **Scaffold(bp)** |
| Total | 113,966 | 529,732,904 | 29,336 | 574,959,875 |
| Max | - | 174,253 | - | 2,042,129 |
| Number>=100 | 113,736 | - | 29,336 | - |
| Number>=2000 | 57,711 | - | 7,801 | - |
| N50 | 12,867 | 10,417 | 750 | 230,234 |
| N60 | 18,770 | 7,765 | 1,035 | 178,368 |
| N70 | 26,816 | 5,575 | 1,412 | 128,609 |
| N80 | 38,421 | 3,718 | 1,967 | 81,082 |
| N90 | 57,494 | 2,014 | 3,079 | 30,247 |

†Only scaffolds with length >= 200 bp were counted.

**Supplementary Table 3.** Assessment of genome coverage rate based on short-insert paired-end reads remapping analysis

|  |  | **Percentage (%)** |
| --- | --- | --- |
| Reads | Mapping rate (%) | 87.4 |
| Genome | Average sequencing depth | 71.98 |
|  | Coverage (%) | 99.74 |
|  | Coverage at least 4X (%) | 98.61 |
|  | Coverage at least 10X (%) | 96.58 |
|  | Coverage at least 20X (%) | 92.92 |

**Supplementary Table 4.** Assessment of gene coverage rate using Trinity assembled sequences (Unigenes)

| **Dataset** | **Number** | **Total length (bp)** | **Sequences covered by assembly (%)** | **With > 90% sequence in one scaffold** | | **With > 50% sequence in one scaffold** | |
| --- | --- | --- | --- | --- | --- | --- | --- |
|  |  |  |  | **Number** | **Percent** | **Number** | **Percent** |
| > 0bp | 18,529 | 21,132,160 | 99.63 | 16,543 | 89.28 | 18,207 | 98.26 |
| > 200bp | 18,529 | 21,132,160 | 99.63 | 16,543 | 89.28 | 18,207 | 98.26 |
| > 500bp | 18,529 | 21,132,160 | 99.63 | 16,543 | 89.28 | 18,207 | 98.26 |
| > 1000bp | 7,325 | 13,371,531 | 99.89 | 6,415 | 87.58 | 7,178 | 97.99 |
| > 2000bp | 2,118 | 6,230,088 | 100.00 | 1,903 | 89.85 | 2,069 | 97.69 |
| > 5000bp | 87 | 554,752 | 100.00 | 74 | 85.06 | 83 | 95.40 |

**Supplementary Table 5** BUSCO evaluation of *R. piscesae* genome assembly

|  | *R. piscesae* |
| --- | --- |
| Complete BUSCOs | 884 |
| Complete and single-copy BUSCOs | 878 |
| Complete and duplicated BUSCOs | 6 |
| Fragmented BUSCOs | 27 |
| Missing BUSCOs | 43 |
| Total BUSCO groups searched | 954 |

**Supplementary Table 6** Summary of annotated repeats in *R. piscesae* genome

|  | Number | Length（bp） | Percentage（%） |
| --- | --- | --- | --- |
| **Retroelements** | **203,600** | **56,094,618** | **9.76** |
| SINEs: | 26,182 | 5,142,860 | 0.89 |
| Penelope | 5,103 | 888,385 | 0.15 |
| LINEs: | 164,545 | 46,431,336 | 8.08 |
| CRE/SLACS | 313 | 32329 | 0.01 |
| L2/CR1/Rex | 106,412 | 29,637,674 | 5.15 |
| R1/LOA/Jockey | 61 | 10,440 | 0.00 |
| R2/R4/NeSL | 394 | 118,283 | 0.02 |
| RTE/Bov-B | 39,673 | 12,731,947 | 2.21 |
| L1/CIN4 | 3,031 | 249,962 | 0.04 |
| LTR: | 12,873 | 4,520,422 | 0.79 |
| BEL/Pao | 33 | 6,420 | 0.00 |
| Ty1/Copia | 8 | 1,185 | 0.00 |
| Gypsy/DIRS1 | 8,533 | 3,448,083 | 0.60 |
| Retroviral | 456 | 24,641 | 0.00 |
| **DNA transposons：** | **35,533** | **10,993,251** | **1.91** |
| hobo-Activator | 14,208 | 3,067,637 | 0.53 |
| Tc1-IS630-Pogo | 2976 | 1,100,786 | 0.19 |
| PiggyBac | 2 | 147 | 0.00 |
| Tourist/Harbinger | 1,511 | 760,090 | 0.13 |
| Other (Mirage,P-element,Transib) | 905 | 214,308 | 0.04 |
| **Rolling circles** | **1,933** | **211,701** | **0.04** |
| **Unclassified:** | **546,050** | **106,343,896** | **18.50** |
| **Small RNA:** | **1,290** | **152,996** | **0.03** |
| **Satellites:** | **547** | **175,801** | **0.03** |
| **Simple repeats:** | **282,603** | **24,492,855** | **4.26** |
| **Low complexity** | **9,714** | **828,375** | **0.14** |

**Supplementary Table 7.** Statistics of functional annotated gene models in the genome of *R. piscesae*

|  | **Number** | **Percentage (%)** |
| --- | --- | --- |
| InterPro | 16,309 | 67.68 |
| GO | 16,741 | 69.48 |
| Pfam | 16,068 | 66.68 |
| Swissprot | 17,849 | 74.07 |
| TrEMBL | 21,721 | 90.14 |
| KEGG | 17,022 | 70.64 |
| **Annotated** | 23,021 | 95.54 |
| **Unannotated** | 1,075 | 4.46 |
| Total | 24,096 | - |

**Supplementary Table 8** Information of genomes used to perform phylogenomic analysis

| **Species name** | **NCBI ID** | **Reference** |
| --- | --- | --- |
| *Lamellibrachia lumyesi* | GCA_009193005.1 | (Li et al., 2019) |
| *Paraescarpia echinospica* | GCA_020002185.1 | (Sun et al., 2021) |
| *Ridgeia piscesae* | - | This study |
| *Riftia pachyptila* | - | (de Oliveira et al., 2022) |
| *Capitella teleta* | GCA_000328365 | (Simakov et al., 2013) |
| *Helobdella robusta* | GCA_000326865.1 | (Simakov et al., 2013) |
| *Eisenia andrei* | - | (Shao et al., 2020) |
| *Lingula anatina* | GCA_001039355.2 | (Luo et al., 2015) |
| *Aplysia californica* | GCA_000002075.2 | - |
| *Octopus bimaculoides* | GCA_001194135 | (Albertin et al., 2015) |
| *Notospermus geniculatus* | GCA_002633025.1 | (Luo et al., 2018) |
| *Lottia gigantea* | GCA_000327385 | (Simakov et al., 2013) |
| *Phoronis australis* | GCA_002633005.1 | (Luo et al., 2018) |
| *Adineta vaga* | GCA_021613535.1 | (Flot et al., 2013) |
| *Echinococcus multilocularis* | GCA_000469725.3 | (Tsai et al., 2013) |

**Supplementary Table 9** Exon and intron lengths of genes in four Vestimentiferan tubeworms

| **Species** | **Genome size (Mb)** | **Total Exon Length (bp)** | **Total Intron Length (bp)** | **Mean Exon Length (bp)** | **Mean Intron Length (bp)** | **Intron / Exon length** |
| --- | --- | --- | --- | --- | --- | --- |
| ***Lamellibrachia luymesi*** | 687.7 | 42,059,392 | 220,083,629 | 204.18 | 1332.65 | 6.53 |
| ***Paraescarpia echinospica*** | 1090.9 | 57,384,225 | 204,708,487 | 375.12 | 1570.63 | 4.19 |
| ***Riftia pachtypila*** | 560.7 | 46,707,225 | 264,839,561 | 229.08 | 1531.29 | 6.68 |
| ***Ridgeia piscesae*** | 574.9 | 36,771,027 | 234,465,512 | 223.80 | 1672.26 | 7.47 |

**Supplementary Table 10** Expression levels of hemoglobin genes with free cysteine in *R. piscesae*

|  | **Gene Name** | **FPKM** |
| --- | --- | --- |
| **A2** | RidPis-gene23569 | 3615.41 |
| **B2** | RidPis-gene18777 | 58691.05 |
| **B1** | RidPis-gene4676 | 1.03 |
|  | RidPis-gene8097 | 160.32 |
|  | RidPis-gene14373 | 15.4 |
|  | RidPis-gene346 | 10.45 |
|  | RidPis-gene14380 | 40.07 |
|  | RidPis-gene21195 | 1.06 |
|  | RidPis-gene22799 | 3887.97 |

**Supplementary Table 11** Gene families were significantly expanded in the genomes of all four tubeworms

|  |  | P. echinospica | L. luymesi | R. piscesae | R. pachyptila | C. teleta | H. robusta | E. andrei | L. gigantea | A. californica | O. bimaculoides | P. australis | N. geniculatus |
| --- | --- | --- | --- | --- | --- | --- | --- | --- | --- | --- | --- | --- | --- |
| OG0000338 | Lysozyme | 12 | 11 | 11 | 7 | 2 | 0 | 9 | 0 | 0 | 2 | 1 | 4 |
| OG0000388 | Globin Domain-Containing Protein | 27 | 10 | 7 | 9 | 0 | 0 | 2 | 0 | 0 | 0 | 0 | 0 |
| OG0000429 | Chitin-Binding Type-4 Domain-Containing Protein | 5 | 6 | 5 | 6 | 1 | 0 | 2 | 9 | 7 | 8 | 2 | 0 |
| OG0000161 | Chitin Binding Peritrophin-A | 31 | 21 | 12 | 19 | 0 | 0 | 0 | 1 | 0 | 0 | 0 | 0 |
| OG0000035 | Chitinase、Chitotriosidase-1 | 24 | 31 | 23 | 18 | 7 | 3 | 4 | 12 | 15 | 15 | 6 | 0 |
| OG0000018 | Lamin-G Domain Protein，Mucin，Chiinese | 55 | 58 | 55 | 15 | 0 | 1 | 4 | 0 | 11 | 8 | 5 | 3 |
| OG0000311 | Glycoprotein-N-Acetylgalactosamine 3-Beta-Galactosyltransferase 1 | 9 | 13 | 10 | 9 | 4 | 2 | 5 | 3 | 3 | 1 | 2 | 0 |
| OG0000282 | C-Type Lectin Perlucin | 17 | 10 | 15 | 7 | 0 | 1 | 2 | 1 | 11 | 0 | 0 | 0 |
| OG0000197 | C-Type Lectin Domain-Containing Receptor 2 | 12 | 14 | 12 | 13 | 9 | 3 | 5 | 1 | 0 | 0 | 3 | 4 |
| OG0000271 | Low Density Lipoprotein Receptor-Related Protein 5-Related | 15 | 16 | 17 | 17 | 0 | 0 | 0 | 0 | 0 | 0 | 1 | 0 |

**Supplementary Table 12** Gene families were significantly expanded in the genomes of two seep-dwelling tubeworms

|  |  | P. Echinospica | L. Luymesi | R. Piscesae | R. Pachyptila | C. Teleta | H. Robusta | E. Andrei | L. Gigantea | A. Californica | O. Bimaculoides | P. Australis | N. Geniculatus |
| --- | --- | --- | --- | --- | --- | --- | --- | --- | --- | --- | --- | --- | --- |
| OG0000601 | Ovochymase-1 | 20 | 15 | 5 | 4 | 0 | 0 | 0 | 0 | 0 | 0 | 0 | 0 |
| OG0000135 | Inhibitor Of Apoptosis | 14 | 16 | 10 | 9 | 8 | 0 | 10 | 9 | 1 | 1 | 3 | 11 |
| OG0000082 | Toll-Like Receptor 4 | 38 | 43 | 21 | 13 | 0 | 0 | 0 | 0 | 0 | 0 | 0 | 0 |
| OG0000222 | Collagen Triple Helix Repeat-Containing Protein 1 | 17 | 36 | 8 | 9 | 0 | 0 | 1 | 0 | 0 | 0 | 0 | 0 |
| OG0000232 | Hemerythrin | 17 | 19 | 9 | 5 | 0 | 18 | 2 | 0 | 0 | 0 | 0 | 0 |
| OG0000560 | Tyrosine-Protein Kinase | 9 | 12 | 8 | 8 | 1 | 0 | 6 | 1 | 0 | 0 | 0 | 0 |
| OG0000632 | Thiosulfate Sulfurtransferase | 6 | 4 | 5 | 4 | 6 | 0 | 0 | 3 | 4 | 2 | 2 | 6 |
| OG0000885 | Poly (Adp-Ribose) Glycohydrolase | 5 | 6 | 4 | 4 | 1 | 1 | 7 | 1 | 1 | 1 | 2 | 2 |
| OG0000272 | Cartilage Intermediate Layer Protein 1 | 27 | 15 | 16 | 8 | 0 | 0 | 0 | 0 | 0 | 0 | 0 | 0 |
| OG0000072 | Protein Mono-Adp-Ribosyltransferase Parp14 | 27 | 35 | 13 | 22 | 1 | 2 | 12 | 0 | 2 | 0 | 6 | 0 |
| OG0000389 | IRE1 | 13 | 26 | 4 | 9 | 0 | 0 | 0 | 0 | 0 | 1 | 1 | 1 |
| OG0000423 | Chitin-Binding Proteins | 26 | 10 | 9 | 7 | 0 | 0 | 0 | 0 | 0 | 0 | 0 | 0 |
| OG0000210 | Cyclic Gmp-Amp Synthase | 16 | 15 | 4 | 10 | 0 | 1 | 27 | 0 | 0 | 0 | 0 | 0 |
| OG0000628 | Deoxynucleoside Triphosphate Triphosphohydrolase Samhd1 | 6 | 8 | 2 | 1 | 3 | 3 | 1 | 3 | 2 | 1 | 4 | 8 |
| OG0000891 | Docking | 10 | 7 | 4 | 5 | 1 | 0 | 2 | 1 | 1 | 1 | 1 | 2 |
| OG0003616 | Netrin Receptor Unc5c | 3 | 13 | 0 | 1 | 0 | 0 | 0 | 0 | 0 | 0 | 0 | 0 |
| OG0000034 | Calcium-Activated Chloride Channel Regulator 3a | 38 | 39 | 18 | 12 | 11 | 0 | 0 | 3 | 2 | 0 | 17 | 21 |
| OG0000048 | Complement Component-Related Sushi Domain-Containing | 47 | 34 | 21 | 19 | 2 | 0 | 0 | 5 | 3 | 1 | 3 | 6 |

**Supplementary Table 13** Positively selected genes (PSGs) in *R. piscesae*

| **No.** | **Gene ID** | **Gene Name** | **Abbreviation** | **FPKM** |
| --- | --- | --- | --- | --- |
| **1** | RidPis-gene207 | Ras-related and estrogen-regulated growth inhibitor | *RERG* | 8.76 |
| **2** | RidPis-gene1338 | AN1-type zinc finger protein 2B | *ZFAND2B* | 0.74 |
| **3** | RidPis-gene7872 | NADH dehydrogenase [ubiquinone] 1 alpha subcomplex subunit 7 | *NDUFA7* | 248.19 |
| **4** | RidPis-gene9582 | alkB homolog 2, alpha-ketoglutarate-dependent dioxygenase | *ALKBH2* | 0.48 |
| **5** | RidPis-gene15769 | Aminoacyl tRNA synthase complex-interacting multifunctional protein 1 | *AIMP1* | 20.93 |
| **6** | RidPis-gene16720 | Rho GTPase-activating protein 6 | *ARHGAP6* | 3.49 |
| **7** | RidPis-gene18649 | Succinate dehydrogenase assembly factor 3 | *SDHAF3* | 19.23 |
| **8** | RidPis-gene20248 | Derlin-1 | *DERL1* | 32.13 |
| **9** | RidPis-gene21655 | Trifunctional enzyme subunit beta, mitochondrial | *HADHB* | 23.88 |

**Supplementary Table 14** Summary of genome assemblies using two other assemblers

|  | **Total length (bp)** | **Number of contigs** | **Contig N50 (bp)** |
| --- | --- | --- | --- |
| **ABySS2** | 464,623,384 | 16,94,402 | 934 |
| **Platanus** | 2,172,372,402 | 15,345,511 | 911 |

**Reference**

Albertin, C.B., Simakov, O., Mitros, T., Wang, Z.Y., Pungor, J.R., Edsinger-Gonzales, E., et al. (2015). The octopus genome and the evolution of cephalopod neural and morphological novelties. *Nature* 524(7564)**,** 220-224. doi: 10.1038/nature14668.

de Oliveira, A.L., Mitchell, J., Girguis, P., and Bright, M. (2022). Novel Insights on Obligate Symbiont Lifestyle and Adaptation to Chemosynthetic Environment as Revealed by the Giant Tubeworm Genome. *Mol Biol Evol* 39(1). doi: 10.1093/molbev/msab347.

Flot, J.F., Hespeels, B., Li, X., Noel, B., Arkhipova, I., Danchin, E.G., et al. (2013). Genomic evidence for ameiotic evolution in the bdelloid rotifer Adineta vaga. *Nature* 500(7463)**,** 453-457. doi: 10.1038/nature12326.

Li, Y., Tassia, M.G., Waits, D.S., Bogantes, V.E., David, K.T., and Halanych, K.M. (2019). Genomic adaptations to chemosymbiosis in the deep-sea seep-dwelling tubeworm Lamellibrachia luymesi. *BMC Biol* 17(1)**,** 91. doi: 10.1186/s12915-019-0713-x.

Luo, Y.J., Kanda, M., Koyanagi, R., Hisata, K., Akiyama, T., Sakamoto, H., et al. (2018). Nemertean and phoronid genomes reveal lophotrochozoan evolution and the origin of bilaterian heads. *Nat Ecol Evol* 2(1)**,** 141-151. doi: 10.1038/s41559-017-0389-y.

Luo, Y.J., Takeuchi, T., Koyanagi, R., Yamada, L., Kanda, M., Khalturina, M., et al. (2015). The Lingula genome provides insights into brachiopod evolution and the origin of phosphate biomineralization. *Nat Commun* 6. doi: 10.1038/ncomms9301.

Shao, Y., Wang, X.B., Zhang, J.J., Li, M.L., Wu, S.S., Ma, X.Y., et al. (2020). Genome and single-cell RNA-sequencing of the earthworm Eisenia andrei identifies cellular mechanisms underlying regeneration. *Nat Commun* 11(1)**,** 2656. doi: 10.1038/s41467-020-16454-8.

Simakov, O., Marletaz, F., Cho, S.J., Edsinger-Gonzales, E., Havlak, P., Hellsten, U., et al. (2013). Insights into bilaterian evolution from three spiralian genomes. *Nature* 493(7433)**,** 526-531. doi: 10.1038/nature11696.

Sun, Y., Sun, J., Yang, Y., Lan, Y., Ip, J.C., Wong, W.C., et al. (2021). Genomic signatures supporting the symbiosis and formation of chitinous tube in the deep-sea tubeworm Paraescarpia echinospica. *Mol Biol Evol*. doi: 10.1093/molbev/msab203.

Tsai, I.J., Zarowiecki, M., Holroyd, N., Garciarrubio, A., Sanchez-Flores, A., Brooks, K.L., et al. (2013). The genomes of four tapeworm species reveal adaptations to parasitism. *Nature* 496(7443)**,** 57-63. doi: 10.1038/nature12031.
